# Phylogenetic Diversity and Biological Activity of Actinobacteria Isolated from the Chukchi Shelf Marine Sediments in the Arctic Ocean

**DOI:** 10.3390/md12031281

**Published:** 2014-03-06

**Authors:** Meng Yuan, Yong Yu, Hui-Rong Li, Ning Dong, Xiao-Hua Zhang

**Affiliations:** 1 SOA Key Laboratory for Polar Science, Polar Research Institute of China, Shanghai 200136, China; E-Mail: lihuirong@pric.gov.cn; 2 Department of Marine Biology, Ocean University of China, Qingdao 266003, China; E-Mails: dfsq1989@126.com (M.Y.); dongn.eve@gmail.com (N.D.); xhzhang@ouc.edu.cn (X.-H.Z.)

**Keywords:** marine Actinobacteria, phylogenetic diversity, biological activity, Arctic Ocean

## Abstract

Marine environments are a rich source of Actinobacteria and have the potential to produce a wide variety of biologically active secondary metabolites. In this study, we used four selective isolation media to culture Actinobacteria from the sediments collected from the Chukchi Shelf in the Arctic Ocean. A total of 73 actinobacterial strains were isolated. Based on repetitive DNA fingerprinting analysis, we selected 30 representatives for partial characterization according to their phylogenetic diversity, antimicrobial activities and secondary-metabolite biosynthesis genes. Results from the 16S rRNA gene sequence analysis indicated that the 30 strains could be sorted into 18 phylotypes belonging to 14 different genera: *Agrococcus*, *Arsenicicoccus*, *Arthrobacter*, *Brevibacterium*, *Citricoccus*, *Janibacter*, *Kocuria*, *Microbacterium*, *Microlunatus*, *Nocardioides*, *Nocardiopsis*, *Saccharopolyspora*, *Salinibacterium* and *Streptomyces*. To our knowledge, this paper is the first report on the isolation of *Microlunatus* genus members from marine habitats. Of the 30 isolates, 11 strains exhibited antibacterial and/or antifungal activity, seven of which have activities against *Bacillus subtilis* and *Candida albicans*. All 30 strains have at least two biosynthetic genes, one-third of which possess more than four biosynthetic genes. This study demonstrates the significant diversity of Actinobacteria in the Chukchi Shelf sediment and their potential for producing biologically active compounds and novel material for genetic manipulation or combinatorial biosynthesis.

## 1. Introduction

Actinobacteria are Gram-positive bacteria with high guanosine-cytosine content in their DNA, ranging from 51% to >70% [1]. These bacteria are important microorganisms, because they can produce novel metabolites and other molecules of pharmaceutical importance. Since the discovery of actinomycin, over 10,000 bioactive compounds have been produced by Actinobacteria, representing about 45% of all bioactive microbial secondary metabolites [2]. The probability of finding novel bioactive compounds from terrestrial Actinobacteria is low; thus, some scholars have emphasized that marine environments might be a rich source of novel Actinobacteria that could potentially produce interesting new bioactive secondary metabolites [3,4,5]. Molecular ecological studies have revealed that deep-sea sediments contained more than 1300 different actinobacterial operational taxonomic units, most of which were predicted to represent novel species, genera and families [6]. It is not surprising, therefore, that members of 50 genera, including 12 novel genera, of Actinobacteria have been isolated from marine habitats [7]. Meanwhile, structurally unique natural products with biological activities have been increasingly recovered from marine Actinobacteria [7,8,9,10,11,12]. In terms of geographical origins, most of these microorganisms originated from tropical and temperate seas.

Only a few investigations on marine Actinobacteria have been performed in the Arctic marine environment. However, these studies have revealed a diversity of Actinobacteria in the Arctic marine sediments [13,14,15,16,17,18,19], the sea surface microlayer [17,20] and marine sponges [20]. Recently, a novel macrolactam and a new thiopeptide with antibiotic activities have been discovered from Arctic marine sediment-derived Actinobacteria [18,19]. In the present study, we used several selective media to isolate Actinobacteria from the Chukchi Shelf sediments in the Arctic Ocean. A total of 73 actinobacterial isolates were cultivated and partially characterized in terms of phylogenetic diversity, antimicrobial activities and secondary-metabolite biosynthesis genes. The results indicated a significant diversity of Actinobacteria in the Chukchi Shelf sediment and that this habitat can be a suitable source for isolating novel actinomycetes that produce antimicrobial compounds.

## 2. Results and Discussion

### 2.1. Isolation of Actinobacteria from the Chukchi Shelf Marine Sediment

Dispersion and differential centrifugation (DDC) was employed to isolate Actinobacteria from 10 samples of Chukchi Shelf marine sediments collected from 26 m to 69.6 m deep. After one week to four weeks, individual colonies were picked from the isolation media according to morphological characteristics and further isolated on fresh media as pure cultures. A total of 165 strains were isolated from the Chukchi Shelf marine sediments and grown on MISP2. Of all the strains, only 25 isolates formed powdery colonies with well-developed aerial hyphae. The remaining isolates formed white, yellow, orange, pink, red or brown pigmented colonies without aerial hyphae. The partial 16S rRNA gene sequence (~500 nt) analysis confirmed that 73 of the 165 isolates belonged to Actinobacteria. Repetitive DNA fingerprinting analysis was performed to dereplicate the actinobacterial isolates from the same sample. Then, from the 73 actinobacterial isolates, 30 representatives were selected for further investigation. Among the 30 representatives, six strains were obligate marine Actinobacteria that require seawater for growth, and nine strains grew better on the ISP2 media prepared with seawater than on those prepared with deionized water plus 3.2% NaCl (salinity of the seawater) and on those prepared with deionized water (Table 1). These results suggested that a large number of Actinobacteria adapted well to the marine habitats in the Chukchi Shelf sediments.

### 2.2. Phylogenetic Diversity

**Figure 1:**
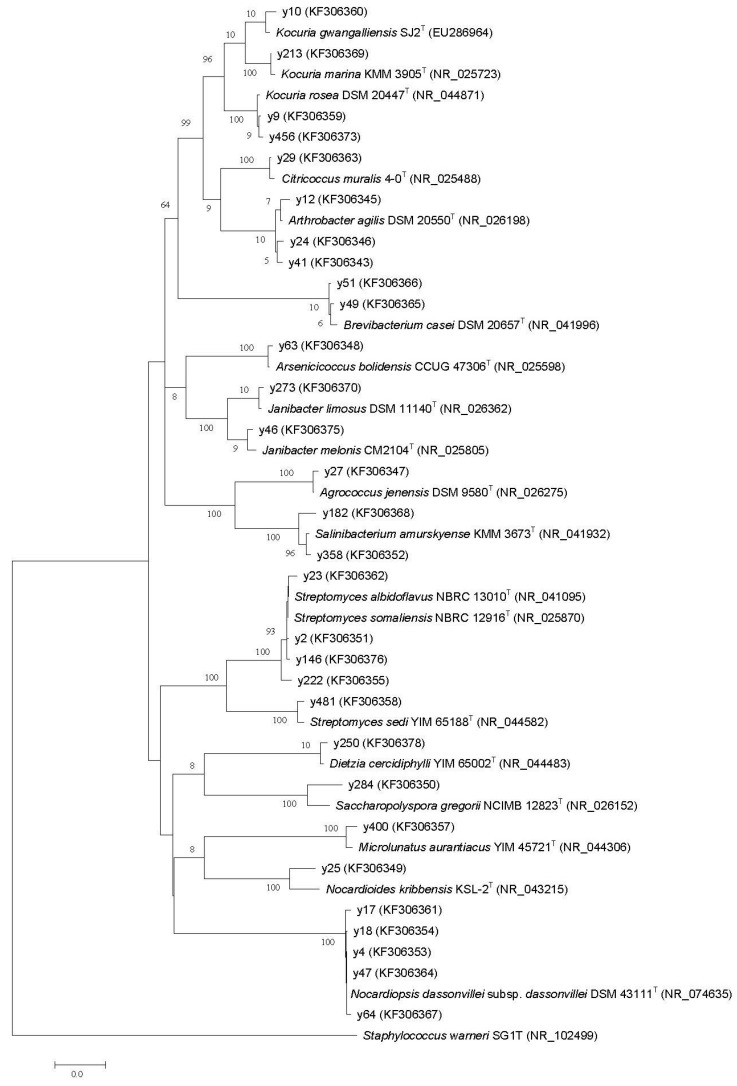
The phylogenetic relationship of the actinobacterial isolates based on 16S rRNA gene homology. The tree was constructed using the neighbor-joining method with Kimura two-state parameters and pairwise-deletion model analyses, which were implemented in the Molecular Evolutionary Genetics Analysis (MEGA), version 5.0 program. The resultant tree topologies were evaluated by bootstrap analysis based on 1000 replicates. The numbers at nodes represent the percentage levels of bootstrap support (%). The GenBank accession numbers of 16S rRNA sequences are given in the parentheses. Bar = 2% sequence divergence.

The nearly complete 16S rRNA gene sequences (1479 bp to 1506 bp) of the 30 representatives were PCR-amplified and sequenced. The results of the 16S rRNA gene sequences analysis are shown in Figure 1 and Table 1. Based on similarity criteria of 98% at the 16S rRNA gene, the 30 isolates were sorted into 18 phylotypes, belonging to 14 different genera, and fell into 10 family level groupings: Brevibacteriaceae, Dietziaceae, Intrasporangiaceae, Microbacteriaceae, Micrococcaceae, Nocardioidaceae, Nocardiopsaceae, Propionibacteriaceae, Pseudonocardiaceae and Streptomycetaceae. Twenty-eight of the 30 isolates shared 99.2% to 99.9% sequence similarities with the closest type strains. The other two strains, y25 and y284, quite likely represented novel species with less than 98% 16S rRNA gene sequence identity to the valid published species [21,22,23]. Interestingly, we found that strain y400 was assigned to the genus, *Microlunatus*, and was most closely related to the type strain of *Microlunatus aurantiacus* YIM 45721^T^ isolated from a rhizosphere soil sample in Yunnan Province, China [24]. To our knowledge, this study is the first to report on the isolation of *Microlunatus* genus members from a marine environment. These data indicated the considerable diversity of Actinobacteria within the Chukchi Shelf sediments.

**Table 1:** Phylogenetic identity, seawater requirement, biosynthetic genes and antimicrobial activities in the actinobacterial isolates.

Genus	Isolate	The Closest Type Strain (% Identity)	Seawater Requirement	Biosynthetic Genes							Antimicrobial Activities *	
				PKS I	PKS II	NRPS	*phz*E	dTGD	Halo	CYP	*B. subtilis*	*C.* *albicans*
Brevibacteriaceae												
*Brevibacterium*	y49	*Brevibacterium casei* NCDO 2048^T^ (99.3)	−	+	−	+	−	+	+	−	−	−
	y51	*Brevibacterium casei* NCDO 2048^T^ (99.4)	−	+	−	+	−	+	−	−	−	−
Dietziaceae												
*Dietzia*	y250	*Dietzia cercidiphylli* YIM 65002^T^ (99.6)	+	+	−	+	−	−	+	−	−	−
Intrasporangiaceae												
*Arsenicicoccus*	y63	*Arsenicicoccus bolidensis* CCUG 47306^T^ (99.8)	−	−	−	−	−	+	+	−	−	−
*Janibacter*	y46	*Janibacter melonis* CM2104^T^ (99.5)	−	+	+	−	−	+	−	−	−	−
	y273	*Janibacter limosus* DSM 11140^T^ (99.7)	−	+	−	+	−	+	+	+	−	−
Microbacteriaceae												
*Agrococcus*	y27	*Agrococcus jenensis* DSM 9580^T^ (99.7)	− ^†^	−	+	−	−	+	−	−	−	−
*Salinibacterium*	y182	*Salinibacterium amurskyense* KMM 3673^T^ (99.0)	−	+	−	−	+	+	−	+	−	−
	y358	*Salinibacterium amurskyense* KMM 3673^T^ (99.7)	− ^†^	+	−	−	−	+	−	−	−	−
Micrococcaceae												
*Arthrobacter*	y12	*Arthrobacter agilis* DSM 20550^T^ (99.5)	−	+	−	+	−	+	−	−	−	−
	y24	*Arthrobacter agilis* DSM 20550^T^ (99.3)	− ^†^	+	−	−	−	+	−	+	−	−
	y41	*Arthrobacter agilis* DSM 20550^T^ (99.2)	− ^†^	+	+	−	−	−	+	+	−	−
*Citricoccus*	y29	*Citricoccus muralis* 4-0^T^ (99.7)	− ^†^	+	−	−	−	+	+	+	−	−
*Kocuria*	y9	*Kocuria rosea* DSM 20447^T^ (99.7)	+	+	−	−	−	−	+	−	−	−
	y456	*Kocuria rosea* DSM 20447^T^ (99.7)	−	+	−	+	−	+	+	−	−	−
	y10	*Kocuria gwangalliensis* SJ2^T^ (99.4)	− ^†^	+	−	−	−	−	+	−	−	−
	y213	*Kocuria marina* KMM 3905^T^ (99.9)	−	+	−	−	+	−	−	−	−	−
Nocardioidaceae												
*Nocardioides*	y25	*Nocardioides kribbensis* KSL-2^T^ (97.8)	+	−	−	−	−	+	+	−	−	−
Nocardiopsaceae												
*Nocardiopsis*	y4	*Nocardiopsis dassonvillei* subsp. *dassonvillei* DSM 43111^T^ (ABUI01000017)	+	−	+	+	+	+	−	−	+	−
	y17	*Nocardiopsis dassonvillei* subsp. *dassonvillei* DSM 43111^T^ (99.7)	− ^†^	−	+	+	−	+	+	−	+	+
	y18	*Nocardiopsis dassonvillei* subsp. *dassonvillei* DSM 43111^T^ (99.8)	+	−	+	+	+	+	−	−	+	+
	y47	*Nocardiopsis dassonvillei* subsp. *dassonvillei* DSM 43111^T^ (99.9)	− ^†^	−	+	−	+	+	−	−	+	−
	y64	*Nocardiopsis dassonvillei* subsp. *dassonvillei* DSM 43111^T^ (99.8)	− ^†^	−	+	−	−	+	−	−	+	+
Propionibacteriaceae												
*Microlunatus*	y400	*Microlunatus aurantiacus* YIM 45721^T^ (99.2)	−	+	−	−	−	+	+	+	+	−
Pseudonocardiaceae												
*Saccharopolyspora*	y284	*Saccharopolyspora gregorii* NCIMB 12823^T^ (97.8)	−	−	+	−	−	+	−	−	−	−
Streptomycetaceae												
*Streptomyces*	y2 *	*Streptomyces somaliensis* NBRC 12916^T^ (99.9)	−	+	−	+	−	+	−	+	+	+
	y23	*Streptomyces somaliensis* NBRC 12916^T^ (99.7)	−	+	−	+	−	−	−	+	+	+
	y146 *	*Streptomyces somaliensis* NBRC 12916^T^ (99.9)	−	+	−	−	+	+	−	+	+	+
	y222	*Streptomyces albidoflavus* DSM 40455^T^ (99.5)	−	+	+	+	−	+	−	+	+	+
	y481	*Streptomyces sedi* YIM 65188^T^ (99.4)	+	+	+	+	−	+	−	−	+	−

^†^ Grew better on ISP2 media prepared with seawater than on those prepared with deionized water plus 3.2% NaCl and on those prepared with deionized water; * y146 showed anti-*E*. *coli* activity; y2 showed anti-*S*. *aureus* activity; no isolates showed activities against Gram-negative bacterium *Pseudomonas aeruginosa*; +, positive; −, negative.

### 2.3. Antimicrobial Activities and Detection of Biosynthetic Genes

Actinobacteria are well known for their ability to produce secondary metabolites, many of which are active against pathogenic microorganisms. Some of the closest neighbors of our isolates were found to produce biologically active compounds. *Dietzia cercidiphylli* was found to produce biosurfactants [25]. Helquinoline, a new tetrahydroquinoline antibiotic, was isolated from *Janibacter melonis* [26]. Two strains of *Arthrobacter agilis* produced potent antibacterial compounds with activity against Gram-positive bacteria and possibly related to novel cyclic thiazolyl peptides [27]. Fu *et al*. found that *Nocardiopsis dassonvillei* subsp. *dassonvillei* could produce α-pyrones [28]. *Streptomyces albidoflavus* was found to produce antifungal compounds, such as a non-polyene substance [29], Antimycin A18 [30], *etc*. The 30 selected actinobacterial isolates obtained from the Chukchi Shelf sediments, which belong to 14 actinobacterial genera, were tested for antimicrobial activities against Gram-positive bacteria *Staphylococcus aureus* and *Bacillus subtilis*, Gram-negative bacteria *Escherichia coli* and *Pseudomonas aeruginosa* and the yeast, *Candida albicans*. The results are presented in Table 1. Of the 30 isolates, 11 exhibited antibacterial and/or antifungal activity. Among the 11 antimicrobial isolates, seven showed activity against both Gram-positive bacterium *Bacillus subtilis* and yeast *Candida albicans*. Only one isolate, y146, and one isolate, y2, had activity against Gram-negative *E. coli* and Gram-positive bacteria *Staphylococcus aureus*, respectively. Furthermore, the data clearly show that strains that can produce antibiotic compounds belonging to three genera: *Streptomyces*, *Nocardiopsis* and *Microlunatus*.

A PCR-based screening for the presence of genes associated with biosynthetic pathways can rapidly predict the unknown strains that have the potential to produce secondary metabolites. Therefore, in parallel with antimicrobial activity testing, we attempted the PCR-based approach to detect type I polyketide synthase (PKS I), PKS II, nonribosomal peptide synthase (NRPS), aminodeoxyisochorismate synthase (*phz*E), dTDP-glucose-4, 6-dehydratase (dTGD), halogenase (Halo) and cytochrome P450 hydroxylase (CYP) genes. Several amplified gene fragments were cloned and sequenced, and subsequent analysis confirmed that these genes encode parts of the expected biosynthetic enzymes [31]. Thus, all isolates have at least two secondary-metabolite biosynthetic genes, and one-third of the isolates possess more than four biosynthetic genes (Table 1). The number of strains that exhibited a certain gene with the corresponding number of phylotypes they represent are as follows: PKS I loci, 21 (70%) strains and 13 phylotypes; PKS II loci, 11 (36.7%) strains and seven phylotypes; NRPS loci, 13 (43.3%) strains and eight phylotypes; *phz*E loci, six (20%) strains and four phylotypes; dTGD loci, 24 (80%) strains and 15 phylotypes; Halo loci, 12 (40%) strains and 11 phylotypes; and CYP loci, 10 (33.3%) strains and six phylotypes. These results suggested that the 6-deoxyhexoses glycosylation biosynthetic and type I PKS pathways were widespread in the Chukchi Shelf sediment-derived Actinobacteria. We successfully amplified secondary metabolite biosynthetic gene fragments from the genomes of these isolates with detectable antimicrobial activity. However, most of the non-active isolates have at least one kind of PKS I, PKS II, NRPS and *phz*E gene involved in the backbone biosynthesis of secondary metabolites and one of the dTGD and Halo genes encoding modification enzymes responsible for the introduction and generation of diversity and activity in several secondary metabolites [32,33]. The CYP gene, which encodes the key enzyme, cytochrome P450 hydroxylase, in polyene antibiotics biosynthesis [34] could be detected in several strains of *Janibacter*, *Salinibacterium* and *Arthrobacter* genera without antimicrobial activity. We also sequenced the PCR products of the ketosynthase (KS) domain of PKS II from five *Nocardiopsis* strains genomes. The KS sequences from these strains displayed the amino acid sequence identities (67% to 69%) of the KS domain of fabF involved in the biosynthesis of pradimicin, an antifungal antibiotic from *Actinomadura hibisca* [35], or had 68% similarity with TamM, which is related to the biosynthesis of Tetarimycin A, a tetracyclic methicillin-resistant *Staphylococcus aureus* (MRSA)-active antibiotic [36]. These results revealed that the Actinobacteria associated with the sediments of the Chukchi Shelf possess the potential ability to produce diversely bioactive secondary metabolites. We will attempt to use a variety of media and culture conditions to induce the expression of secondary metabolite gene clusters in these isolates. Other bioactivities of the isolates, e.g., anticancer activity or immunosuppressive activities, will also be analyzed. Furthermore, in contrast to the genus, *Streptomyces*, only a limited number of secondary metabolite biosynthesis gene clusters in rare Actinobacteria have been studied [37]. The biosynthetic gene, such as dTGD or Halo, from our rare actinobacterial isolates may provide novel material for genetic manipulation and combinatorial biosynthesis, which may lead to the generation of pharmaceutical compounds.

## 3. Experimental Section

### 3.1. Sediments Sample

**Figure 2:**
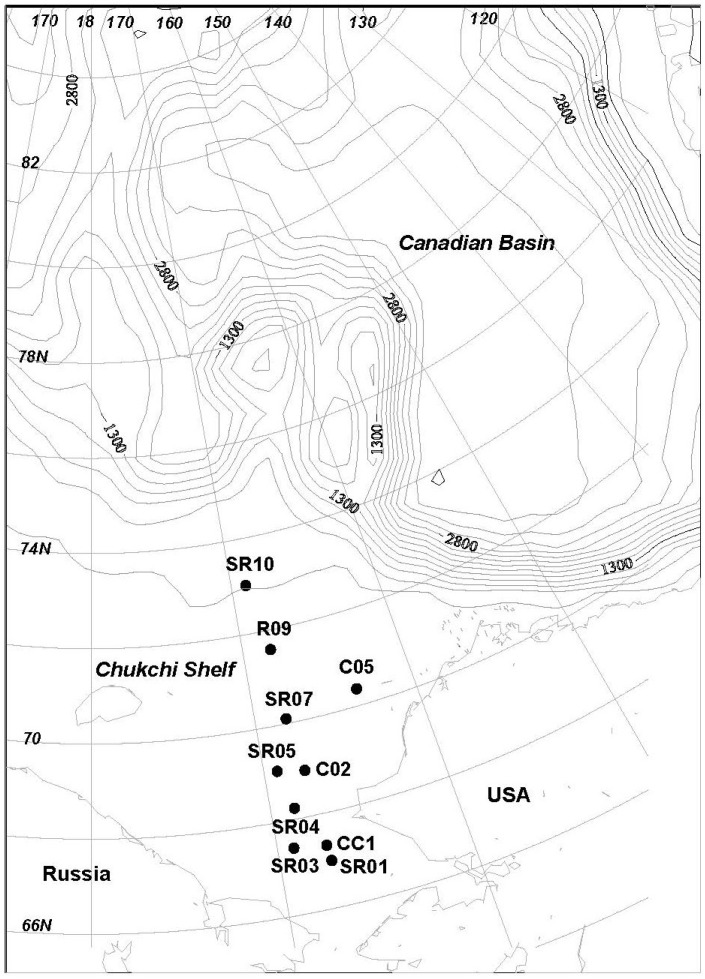
Sediment sampling locations.

A total of 10 sediment samples were collected during the 4th Chinese National Arctic Research Expedition cruise of the icebreaker, Xue Long, into the Arctic Ocean during the summer of 2010 (Figure 2, Table 2). The sediments were sampled using the Ekman-bottom grab. Only the upper 3 cm of the sediment were stored in sterilized 500 mL plastic zip-lock bags and transported to the laboratory at temperatures between 0 °C and 4 °C.

**Table 2:** Characteristics of Chukchi Shelf sediments in the Arctic Ocean.

Samples	Water Depth (m)	Color	Composition	Benthos
CC1	45	Light grey	Silty clay	Bivalves
C02	41	Grey	Clay	Conch
C05	26	Cinerous	Fine sand	Crab, conch, sand dollar, Polychaeta
SR01	41.5	Cinerous	Silty sand	Conch, bivalve
SR03	50.7	Cinerous	Clay	Polychaeta, conch, sipunculoid
SR04	48	Grey	Silty clay	None
SR05	46.7	Cinerous	Silty clay	Conch
SR07	29.7	Cinerous	Silty mud	None
R09	43.5	Grey	Mud soil	None
SR10	69.6	Cinerous	Silty clay	Sea star, conch

### 3.2. Isolation of Actinobacterial Strains

The sediment samples were treated through DDC [38] and a water bath at 55 °C for 6 min. An aliquot of 200 μL of the pretreated 10^0^, 10^−1^ and 10^−2^ dilution was spread over the surface of the isolation media in Petri dishes. The following four isolation media were used: 

**M1:** 0.4 g peptone, 2 g yeast extract, malt extract 2 g, 15 g agar and 1 L sea water.

**SC:** 10 g soluble starch, 1 g casein, 2 g dipotassium phosphate, 0.02 g calcium carbonate, 2 g potassium nitrate, 2 g sodium chloride, 0.05 g magnesium sulfate heptahydrate, 0.01 g ferrous sulfate heptahydrate, 15 g agar and 1 L sea water.

**ISP2:** 4 g glucose, 4 g yeast extract, 10 g malt extract, 15 g agar and 1 L sea water.

**MR2A:** 0.5 g yeast extract, 0.5 g peptone, 0.5 g casein hydrolysate, 0.5 g glucose, 0.5 g soluble starch, 0.3 g sodium pyruvate, 15 g agar and 1 L sea water.

All isolation media were prepared with 1 L natural seawater and amended with filtered (0.2 μm pore size) nalidixic acid (25 μg/mL), nystatin (50 μg/mL) and cycloheximide (50 μg/mL) to inhibit the growth of Gram-negative bacteria and fungi. The isolation plates were incubated in the dark at 28 °C for 4 weeks. Colonies from various agar plates were selected based on differing colony morphologies. Isolates were obtained in pure culture after three successive transfers to fresh isolation media. Pure cultures were frozen in 20% glycerol at −80 °C for long-term storage.

### 3.3. Dereplication by Rep-Polymerase Chain Reaction (PCR)

Repetitive DNA fingerprinting was performed on the actinobacterial isolates from the same sample. Genomic DNA was prepared using a bacterial genomic DNA kit (TIANGENE, Beijing, China), according to the supplied protocol from each isolate. PCR amplification was performed using the BOXA1R primer (5′-CTA CGG CAA GGC GAC GCT GAC G-3′) [33]. The PCR reaction mixture (25 μL) contained: 1 μL genomic DNA extract, 2.5 μL 10× buffer, 2 μL deoxynucleotide triphosphate (dNTP) mixture (2.5 mM), 2 μL BOXA1R primer (25 μM) and 2 U Taq DNA polymerase. The reaction started with pre-denaturation at 95 °C for 8 min followed by 30 cycles of denaturation at 95 °C for 1 min, annealing at 53 °C for 1 min and 4 min of extension at 65 °C, with a final extension at 65 °C for 8 min. PCR products were separated by 2% agarose gel electrophoresis. The gels were analyzed using the program, Quantity one (Bio-Rad). The similarity between patterns was calculated using Pearson coefficients. The clustering pattern was generated using the unweighted pair group method with the arithmetic mean algorithm. If the strains formed a cluster defined at the 72% similarity level, they would be assigned to a single species [39,40].

### 3.4. Seawater Requirements

The dereplicated actinobacterial isolates were tested for the required seawater for growth. Frozen stored strains were inoculated onto the ISP2 agar medium prepared using seawater (MISP2). Once sufficient growth had occurred, a sterile wire loop was used to transfer cell material onto a new plate of the same medium prepared with seawater, a plate prepared with deionized water plus 3.2% NaCl and a plate prepared with deionized water. The plates were incubated at 28 °C for up to 4 weeks. Growth was monitored under a stereomicroscope at 64-fold magnification. If no growth was observed on the plate prepared with deionized water, that strain was considered to require seawater for growth.

### 3.5. 16S rRNA Gene Amplification and Phylogenetic Analysis

The 16S rRNA gene was amplified by PCR using primers 8F (5′-AGA GTT TGA TCC TGG CTC AG-3′) and 1492R (5′-GGT TAC CTT GTT ACG ACT T-3′) [41]. The reaction mixture (50 μL) contained: 2 μL genomic DNA extract, 5 μL 10× buffer, 4 μL BSA (1 mg/mL), 1 μL dNTP mixture (2.5 mM), 1 μL each primer (10 μM) and 3.5 U Taq DNA polymerase 3.5 U. PCR conditions were as follows: 6 min at 94 °C followed by 35 cycles of 45 s at 94 °C, 45 s at 55 °C and 1.5 min at 72 °C, followed by an 8-min extension at 72 °C. PCR amplification was performed in an Eppendorf Mastercycler Gradient. An ABI PRISM 3730 sequencer at Shanghai Majorbio Company was used to sequence the PCR products. The identification of phylogenetic neighbors and the calculation of pairwise 16S rDNA sequence similarities were achieved using the EzTaxon-e server [42,43]. In addition, the sequences were compared to the sequences within the NCBI database [44] using the BLASTN algorithm. Sequences were aligned, and a phylogenetic tree was constructed using the Molecular Evolutionary Genetics Analysis (MEGA) software, version 5.0 [45]. DNA sequences were deposited in GenBank under accession numbers: KF306343, KF306345–KF306355, KF306357–KF306370, KF306373, KF306375, KF306376 and KF306378.

### 3.6. Antimicrobial Activity Testing by Agar Diffusion (Inhibition Zones)

Actinobacterial isolates were transferred to the plates containing three different production media: PM3 (20 g oatmeal, 2.5 g glycerol, 0.1 mg FeSO_4_·7H_2_O, 0.1 mg MnCl_2_·4H_2_O, 0.1 mg ZnSO_4_·7H_2_O, 15 g agar and 1 L sea water) [15], PM4 (0.5 g glucose, 2.5 g glycerol, 5.0 g oatmeal, 5.0 g soybean meal, 0.5 g yeast extract, 2.0 g casamino acids, 10 g agar and 1 L sea water) [15] and GYM4 (10 g glucose, 4 g yeast extract, 4 g malt extract, 15 g agar, 1 L sea water and pH 7.2) [46]. The plates were incubated at 28 °C for 1 week to 4 weeks, depending on the growth rate of the isolates. Then, the agar blocks (diameter: 8 mm) that contained actinobacterial cells were excised and placed on the assay plates spread with test microbial strains. Two Gram-positive bacterial strains, *Staphylococcus aureus* ATCC 29213 and *Bacillus subtilis* CMCC 63501, two Gram-negative bacterial strains, *Escherichia coli* ATCC 44102 and *Pseudomonas aeruginosa* ATCC 27853, and one yeast strain, *Candida albicans* ATCC 10231, were used as test strains. The antimicrobial activities were expressed as inhibition zones after 16 h of incubation at 28 °C.

### 3.7. Amplification of Biosynthetic Gene Fragments

To amplify the genes for the KS domains of PKS I, the KS domains of PKS II, the adenylation domains of NRPS, the enzyme PhzE of the phenazine pathways, the enzyme dTGD of 6-deoxyhexoses glycosylation pathway, the enzyme Halo of halogenation pathway and the enzyme CYP in polyene polyketide biosynthesis, the degenerate primers as described previously were used (Table 3). The components and reaction conditions of the PCR mixture are as follows: 

**PKS I** (50 μL): 2 μL template, 5 μL 10× buffer, 2.5 μL BSA (1 mg/mL), 2 μL dNTP (2.5 mM), 1 μL each primer (10 μM) and 2.5 U Taq DNA polymerase; 5 min at 95 °C, followed by 35 cycles of 1 min at 95 °C, 1 min at 60 °C and 2 min at 72 °C, followed by a 5-min extension at 72 °C.

**PKS II** (50 μL): 2 μL template, 5 μL 10× buffer (Mg^2+^ free), 2 μL MgCl_2_ (25 mM), 4 μL BSA (1 mg/mL), 4 μL dNTP (2.5 mM), 5 μL each primer (10 μM) and 2.5 U Taq DNA polymerase; 5 min at 95 °C, followed by 40 cycles of 1 min at 95 °C, 1 min at 64 °C and 1.5 min at 72 °C, followed by a 15-min extension at 72 °C. 

**NRPS** (50 μL): 2 μL template, 5 μL 10× buffer, 2.5 μL BSA (1 mg/mL), 2 μL dNTP (2.5 mM), 1 μL each primer (25 μM) and 2.5 U Taq DNA polymerase; 5 min at 95 °C, followed by 35 cycles of 30 s at 95 °C, 2 min at 59 °C and 4 min at 72 °C, followed by a 10-min extension at 72 °C.

***phz*E** (25 μL): 1 μL template, 2.5 μL 10× buffer, 2 μL BSA (1 mg/mL), 2 μL dNTP (2.5 mM), 0.5 μL each primer (10 μM) and 1 U Taq DNA polymerase; 2 min at 94 °C, followed by 36 cycles of 1 min at 94 °C, 1 min at 54.7 °C and 2 min at 72 °C, followed by a 7-min extension at 72 °C. 

**dTGD** (20 μL): template 0.7 μL, 10× buffer 2.5 μL, BSA (1 mg/mL) 2 μL, dNTP (2.5 mM) 2 μL, each primer (10 μM) 1.5 μL and 3.5 U Taq DNA polymerase; 4 min at 98 °C, followed by 30 cycles of 40 s at 96 °C, 40 s at 50 °C and 1.5 min at 72 °C, followed by a 5-min extension at 72 °C. 

**Halo** (50 μL): template 2 μL, 10× buffer 5 μL, BSA (1 mg/mL) 2.5 μL, dNTP (2.5 mM) 2 μL, each primer (25 μM) 1 μL and 2.5 U Taq DNA polymerase; 3 min at 94 °C, followed by 30 cycles of 1 min at 94 °C, 1.5 min at 58 °C and 1 min at 72 °C, followed by a 5-min extension at 72 °C.

**CYP** (50 μL): 1 μL template, 5 μL 10× buffer (Mg^2+^ free), 2 μL MgCl_2_ (25 mM), 5 μL BSA (1 mg/mL), 4 μL dNTP (2.5 mM), 1 μL each primer (20 μM) and 2.5 U Taq DNA polymerase; 5 min at 96 °C, followed by 45 cycles of 1 min at 96 °C, 30 s at 60 °C and 45 s at 72 °C, followed by a 5-min extension at 72 °C.

**Table 3:** Primers of secondary metabolite biosynthetic genes.

Gene	Primer	Length (bp)	Reference
PKS I (KSMA-F, KSMB-R)	5′-TSGCSATGGACCCSCAGCAG-3′ 5′-CCSGTSCCGTGSGCCTCSAC-3′	700	[47]
PKS II (540F, 1100R)	5′-GGITGCACSTCIGGIMTSGAC-3′ 5′-CCGATSGCICCSAGIGAGTG-3′	554	[48]
NRPS (A3F, A7R)	5′-GCSTACSYSATSTACACSTCSGG-3′ 5′-SASGTCVCCSGTSCGGTAS-3′	700	[49]
*Phz*E (phzEf, phzEr)	5′-GAAGGCGCCAACTTCGTYATCAA-3′ 5′-GCCYTCGATGAAGTACTCGGTGTG-3′	450	[50]
Halo (FW, RV)	5′-TTCCCSCGSTACCASATCGGSGAG-3′ 5′-GSGGGATSWMCCAGWACCASCC-3′	500	[32]
dTGD (dTGD-1, dTGD-2)	5′-GSGGSGSSGCSGGSTTCATSGG-3′ 5′-GGGWRCTGGYRSGGSCCGTAGTTG-3′	600	[51]
CYP (PEH-1, PEH-2)	5′-TGGATCGGCGACGACCGSVYCGT-3′ 5′-CCGWASAGSAYSCCGTCGTACTT-3′	350	[34]

## 4. Conclusions

A total of 73 actinobacterial strains have been isolated from the Chukchi Shelf sediments in the Arctic Ocean. We used repetitive DNA fingerprinting analysis to select 30 representatives for further investigation. An unexpected variety of actinomycete genera were isolated. The 30 representatives were sorted into 18 phylotypes belonging to 14 different genera. Moreover, 25 of the 30 representatives are rare actinomycetes. We also screened them for biosynthetic genes and antimicrobial activities. Of the 30 isolates, 11 exhibited antibacterial and/or antifungal activity, seven of which have activities against *Bacillus subtilis* and *Candida albicans*. All representatives have at least two biosynthetic genes, and one-third of these representatives possess more than four biosynthetic genes. The KS sequences from five *Nocardiopsis* strains with antimicrobial activity displayed the amino acid sequence identities (67% to 69%) of the KS domain of fabF involved in the biosynthesis of pradimicin or had 68% similarity with TamM, related to the biosynthesis of Tetarimycin A. This study demonstrates the significant diversity of Actinobacteria in the Chukchi Shelf sediment. The new strains might represent a valuable source of biologically active compounds with antimicrobial activity and genes for their biosynthesis.

## Abbreviations

DDC: Dispersion and Differential Centrifugation
BSA: Albumin from Bovine Serum
NCBI: National Center of Biotechnology Information
BLASTN: Basic Local Alignment Search Tool of Nucleic acid
PKS: PolyKetide Synthase
NRPS: Nonribosomal Peptide Synthase
*phz*E: aminodeoxyisochorismate synthase
dTGD: dTDP-Glucose-4, 6-Dehydratase
Halo: Halogenase
CYP: Cytochrome P450 hydroxylase
FADH: Flavin Adenine Dinucleotide Hydrogen carrier
